# Main complications of hip arthroplasty: pictorial essay

**DOI:** 10.1590/0100-3984.2018.0075

**Published:** 2020

**Authors:** Dair Jocely Enge Júnior, Adham do Amaral e Castro, Eduardo Kaiser Ururahy Nunes Fonseca, Eduardo Baptista, Michel Bayouth Padial, Laercio Alberto Rosemberg

**Affiliations:** 1 Imaging Department, Hospital Israelita Albert Einstein, São Paulo, SP, Brazil.

**Keywords:** Hip joint/physiopathology, Hip joint/diagnostic imaging, Arthroplasty, replacement, hip/diagnostic imaging, Arthroplasty, replacement, hip/adverse effects

## Abstract

Hip arthroplasty is a widely used and successful orthopedic procedure for the treatment of degenerative, inflammatory, or traumatic joint disease. The procedure promotes significant pain relief, as well as recovery of limb function, reduction of disability, and better quality of life. However, there are related complications, which have characteristic imaging aspects. In the present study, we review the literature and exemplify such complications using images obtained at our facility, illustrating the main radiological aspects of complications such as heterotopic ossification, periprosthetic fractures, osteolysis, infection, wear, and dislocation.

## INTRODUCTION

Hip arthroplasty is a widely used orthopedic procedure, promoting a significant reduction in pain, recovery of limb function, and improved quality of life^(1-3)^. However, there are related complications, with characteristic imaging features, all of which should be known to the radiologist^(1-3)^.

The present study sought to review and illustrate the main complications of hip arthroplasty. The following conditions were reviewed and illustrated, with an emphasis on X-rays: heterotopic ossification, periprosthetic fracture, osteolysis, infection, wear, and dislocation.

## TERMINOLOGY

The term hemiarthroplasty refers to the replacement of only the femoral component of the hip joint. In unipolar hemiarthroplasty, the prosthesis head articulates directly with the acetabulum. In bipolar hemiarthroplasty, a prosthetic component is positioned in the native acetabulum to articulate with the prosthetic femoral head, thereby reducing cartilage wear.

**Figure 1:**
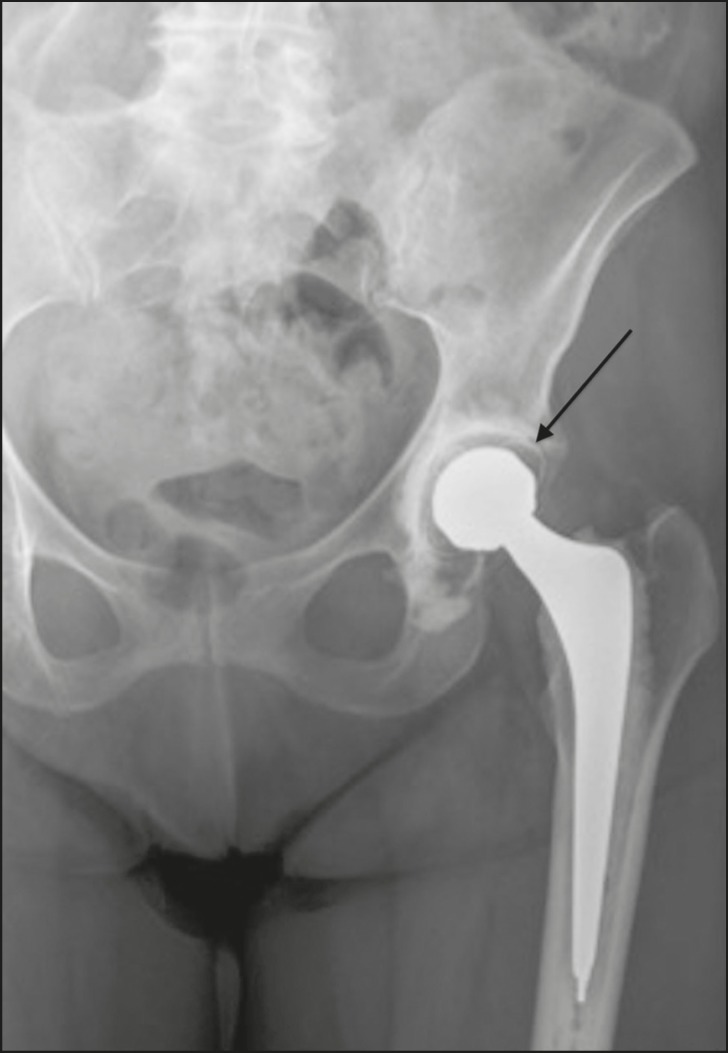
Bipolar total left hip prosthesis (arrow), cemented in the femur and acetabulum, with no evidence of loosening. Polyethylene acetabular component.

The term total arthroplasty is used when the femoral head and acetabulum are both replaced with fixed prosthetic devices. As shown in Figure 1, the prosthetic materials can be fixed with or without cement, either in the femoral or acetabular components^(1-3)^.

## HETEROTOPIC OSSIFICATION

Soft tissue ossification adjacent to prosthetic components is a common complication, occurring in 15-50% of patients undergoing total hip arthroplasty, although it has clinical repercussions in only 1-5% of cases^(1)^. Predisposing factors include male gender, age over 65 years, inflammatory spondyloarthropathies, infections, hip fracture, posttraumatic arthritis, history of heterotopic ossification, and prior hip surgery^(1)^.

Low doses of radiation and of nonsteroidal anti-inflammatory drugs (NSAIDs), which are affordable and readily available, have been useful in the postoperative prophylaxis of heterotopic ossification, the latter being less indicated in patients with gastrointestinal intolerance to NSAIDs or a history of peptic ulcer disease^(2)^.

**Figure 2:**
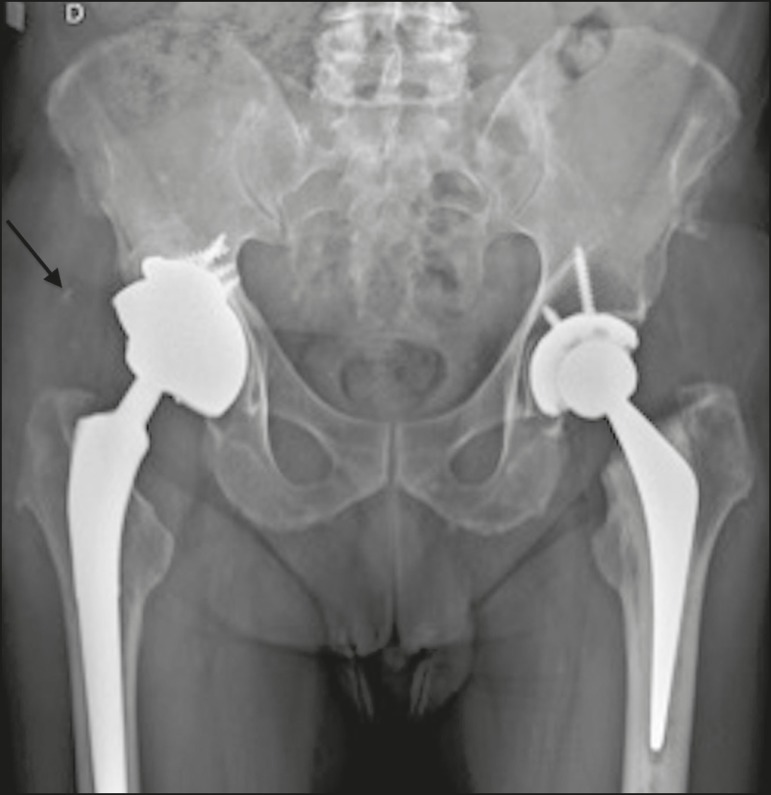
Anteroposterior X-ray of the right hip with grade I heterotopic ossification in the projection of lateral soft tissues on the right (arrow). Total hip arthroplasty in which the acetabular component of the prosthesis was affixed with screws, with no signs of loosening.

**Figure 3:**
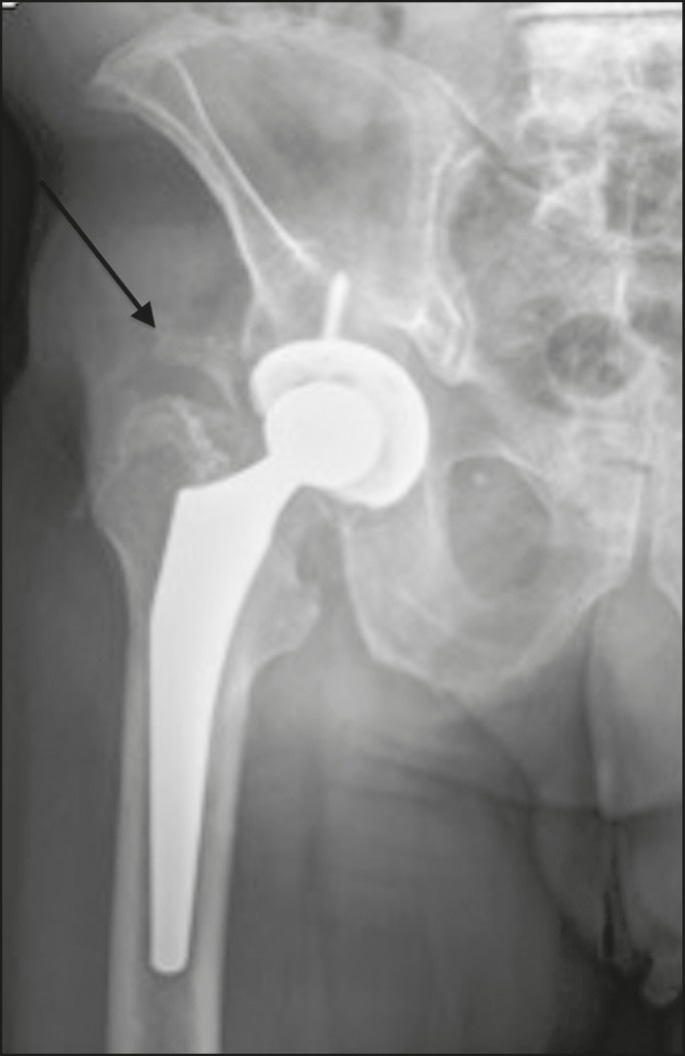
Total right hip arthroplasty in which the prosthesis had an acetabular component with screw fixation and an intramedullary femoral stem, with no signs of loosening or material failure. Grade II peritrochanteric heterotopic ossifications on the right (arrow).

**Figure 4:**
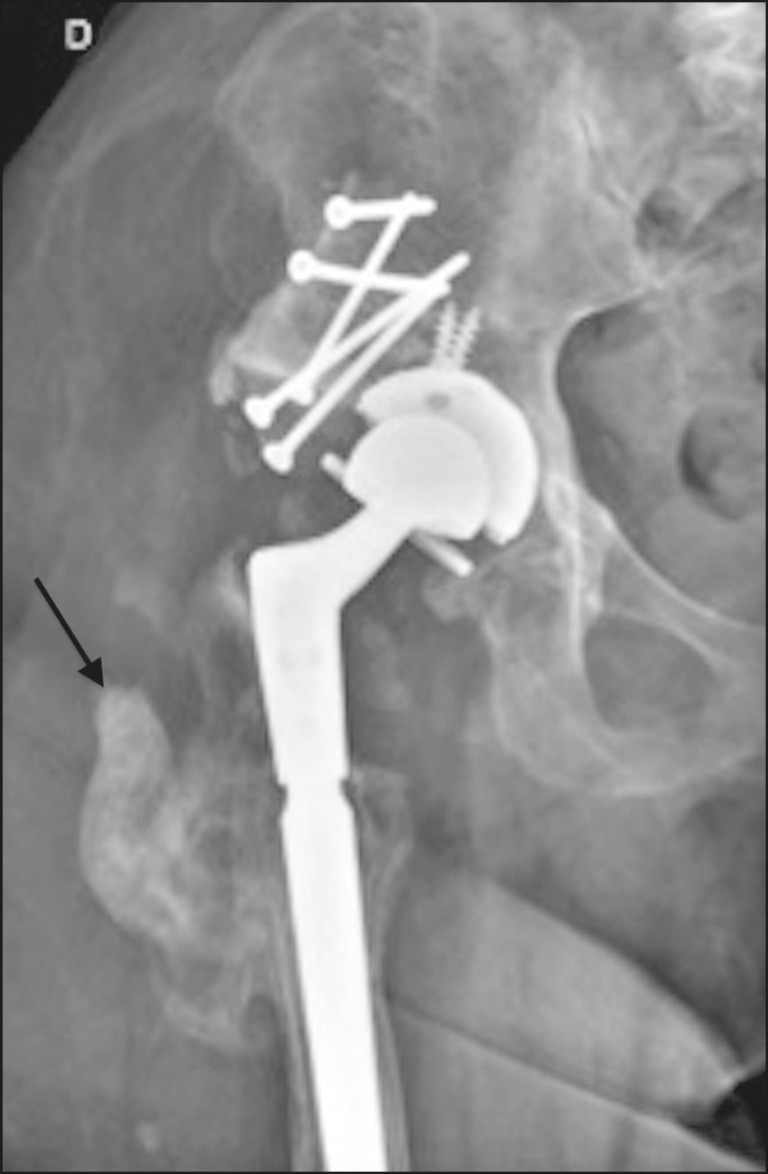
Anteroposterior X-ray of the right hip showing signs of total arthroplasty and bone grafting, with no signs of loosening. Grade III heterotopic ossification of soft tissue near the sites of surgical manipulation (arrow).

**Figure 5:**
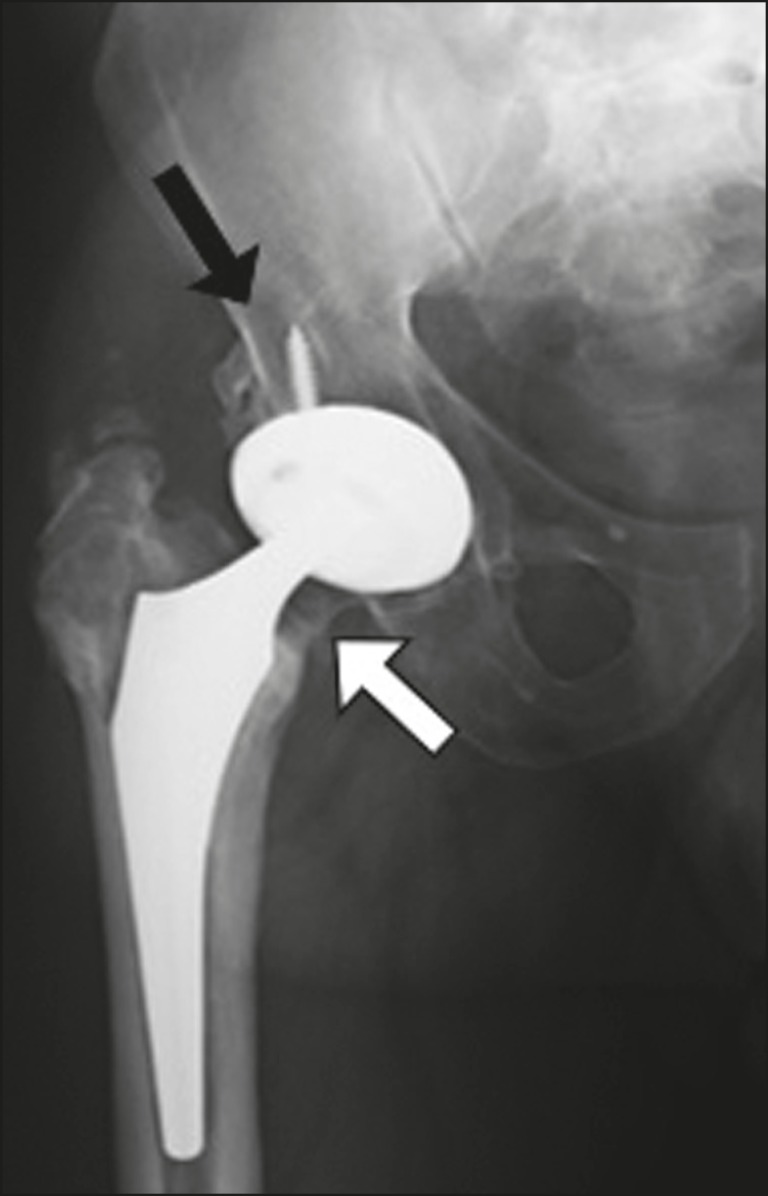
Anteroposterior X-ray of the right hip showing a total bipolar prosthesis and minor resorption (osteolysis—black arrow) of a screw in the acetabular component. Grade IV heterotopic ossification between the femur and the lower border of the acetabulum (white arrow). Cortical thickening without periosteal reaction (stress shielding) is also observed.

Brooker et al.^(3)^classified heterotopic ossification according to the findings on anteroposterior X-rays of the hip: grade 0 = no heterotopic ossification; grade I = one or two foci of heterotopic ossification < 1 cm (Figure 2); grade II = ossification or osteophytes occupying less than half the space between the femur and the pelvis (Figure 3); grade III = ossification or osteophytes occupying more than half of the space between the femur and the pelvis (Figure 4); and grade IV = ossification bridges between the pelvis and femur, indicative of hip ankylosis (Figure 5).

## PERIPROSTHETIC FRACTURE

Periprosthetic fractures occur more frequently around the femoral component than around the acetabular component. Intraoperative femoral fractures can occur during stem placement and are more often associated with uncemented components than with cemented components (in 5.0% vs. 0.3% of cases), due to the high adjustment tension required with uncemented stems^(4)^. They usually occur in the peritrochanteric region, and it is necessary to know their precise location, as well as their relationship with the tip of the metal stem, both of which determine how their treatment will be managed^(5)^. Intraoperative femoral fractures tend to be more common during revisions of hip arthroplasty (7.8%) than during primary arthroplasty (1%), which can be explained by the progression of osteoporosis^(4)^. Postoperative femoral fracture can occur at any time after surgery and is mainly related to traumatic events, typically occurring at the end of the femoral stem, due to “stress risers” that occur at that level, given the difference in rigidity between the metal stem and the adjacent bone^(4,5)^.

**Figure 6:**
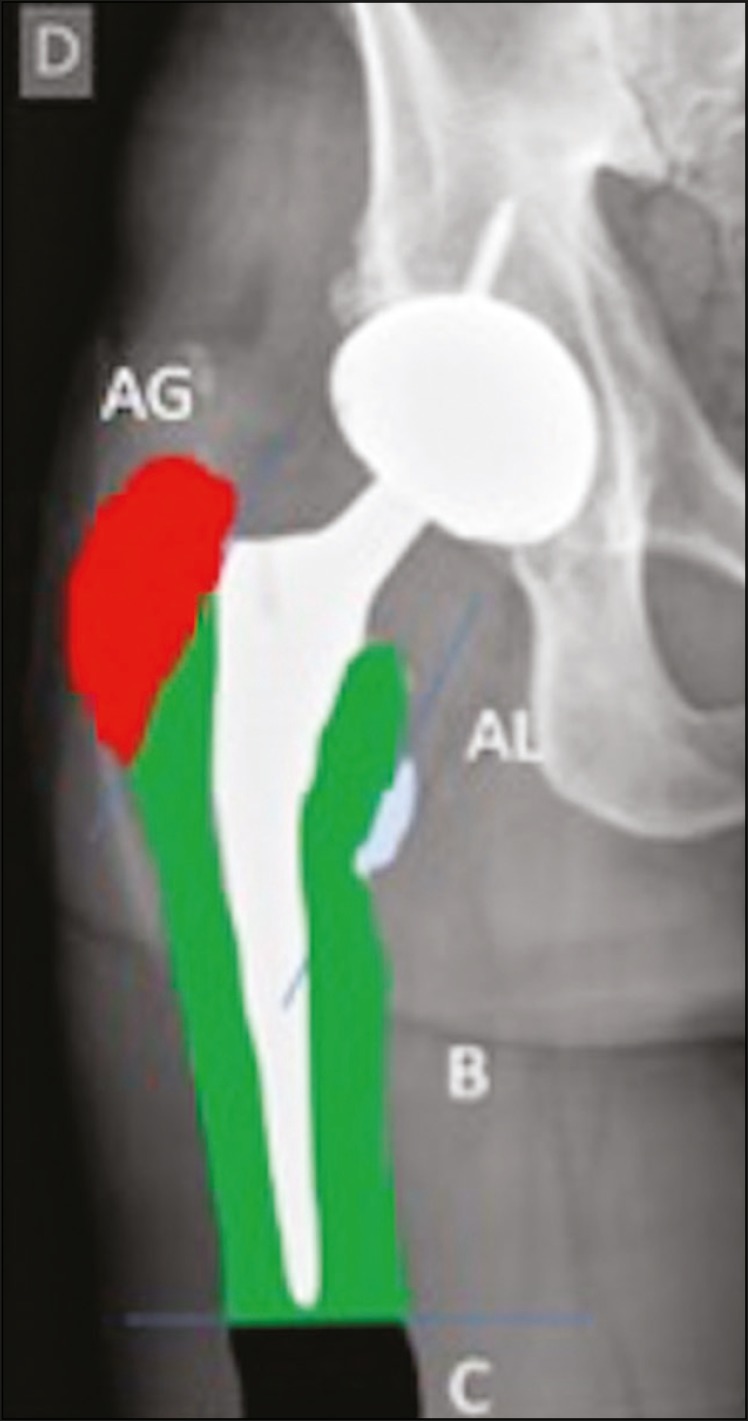
Vancouver classification for periprosthetic fractures.

According to the Vancouver classification, introduced by Duncan et al.^(6)^, which is based on the fracture location (Figure 6), the stability of the metal femoral stem, and the degree of bone loss in the proximal femur, periprosthetic fractures can be divided into three types: type A (peritrochanteric) fractures; type B fractures (those occurring around or just below the tip of the femoral stem); and type C fractures (those occurring well below the implant).

**Figure 7:**
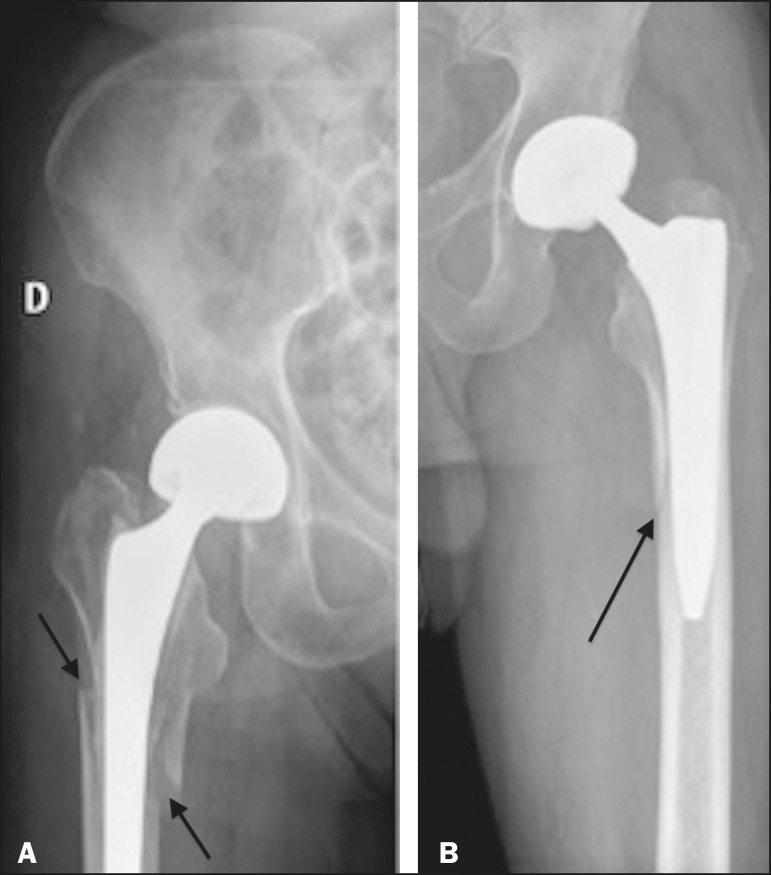
**A:** Anteroposterior X-ray of the right hip showing periprosthetic fracture (arrows) in the femoral component (Vancouver subtype B1). **B:** Anteroposterior X-ray of the left hip showing cortical fracture (arrow) at the medial margin of the femoral prosthetic component (Vancouver subtype B1).

**Figure 8:**
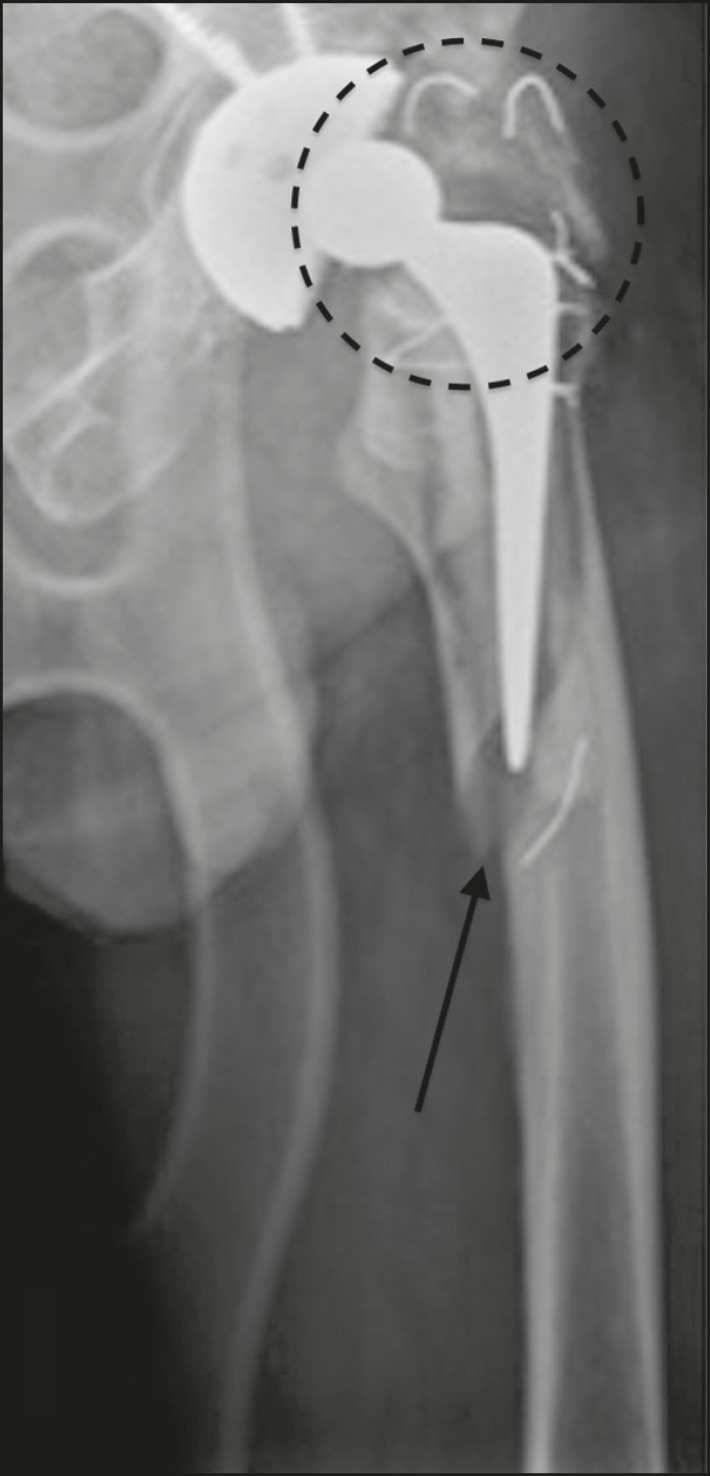
Anteroposterior X-ray of the left hip identifying periprosthetic fracture near the distal femoral stem (arrow), with signs of prosthesis instability (Vancouver subtype B2). Fracture of the greater trochanter with breaks in the metal bands (dotted circle).

The type A periprosthetic fractures are subdivided into the AG subtype (those of the greater trochanter) and the AL subtype (those of the lesser trochanter). The type B fractures are subdivided into the B1 subtype (stable stem; Figure 7), B2 subtype (loose stem; Figure 8), and B3 subtype (loose implant with substantial bone loss). The type C fractures have no subtypes.

## OSTEOLYSIS/INFECTION

**Figure 9:**
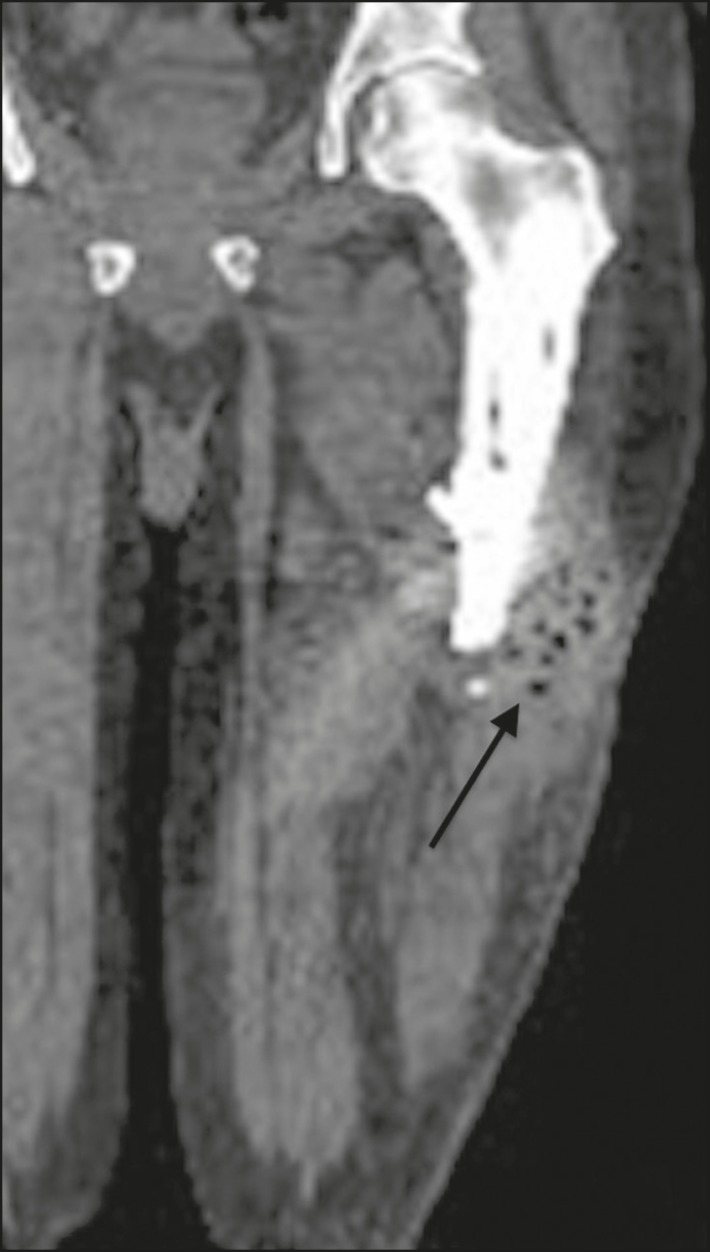
Coronal computed tomography scan of a patient with an infected left femoral prosthesis. Oval formation (arrow), measuring 9.1 × 2.3 cm, containing gas bubbles, located in the middle third of the left thigh, in contact with the femoral stem of the prosthesis, suggestive of abscess.

Foci of periprosthetic lucency that are greater than 2.0 mm or are expanding progressively are signs of abnormality. Making the differential diagnosis between septic and aseptic loosening/osteolysis can be challenging, especially if there are no previous X-rays available for comparative analysis. However, the presence of femoral periosteal reaction or rapidly progressive disease is indicative of septic loosening^(7,8)^. Joint effusion can also be indicative of infection^(7)^, as can periprosthetic fluid collections (Figure 9).

The radiographic appearance of loosening in a cemented prosthesis is a > 2.0 mm lucency at the cement-bone interface, progressive expansion of such a lucency, or even cracking of the cement. In an uncemented prosthesis, loosening manifests as a > 2.0 mm lucency at the metal-bone interface, progressive expansion of such a lucency, or subsidence that is greater than 1.0 cm or is still increasing more than one year after the procedure^(7,8)^.

**Figure 10:**
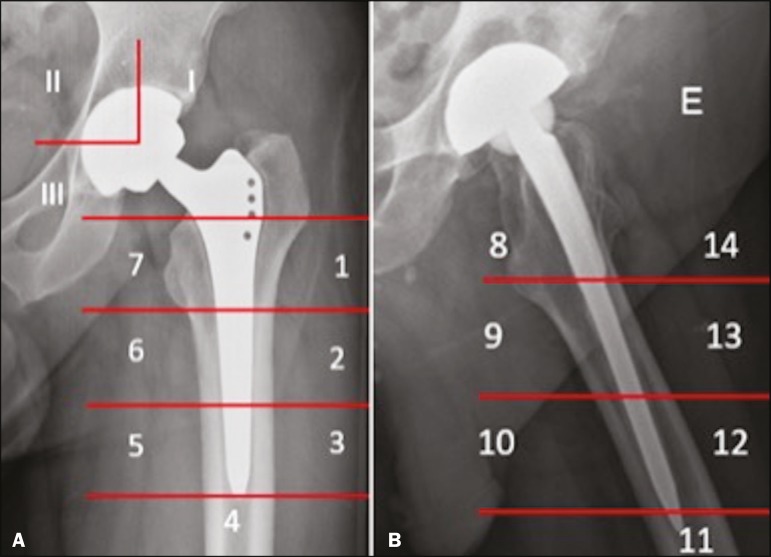
Femoral and acetabular zones in the evaluation of osteolysis. **A:** Anteroposterior X-ray showing the first seven femoral zones and the three acetabular zones. **B:** Lateral X-ray showing the seven additional femoral zones.

**Figure 11:**
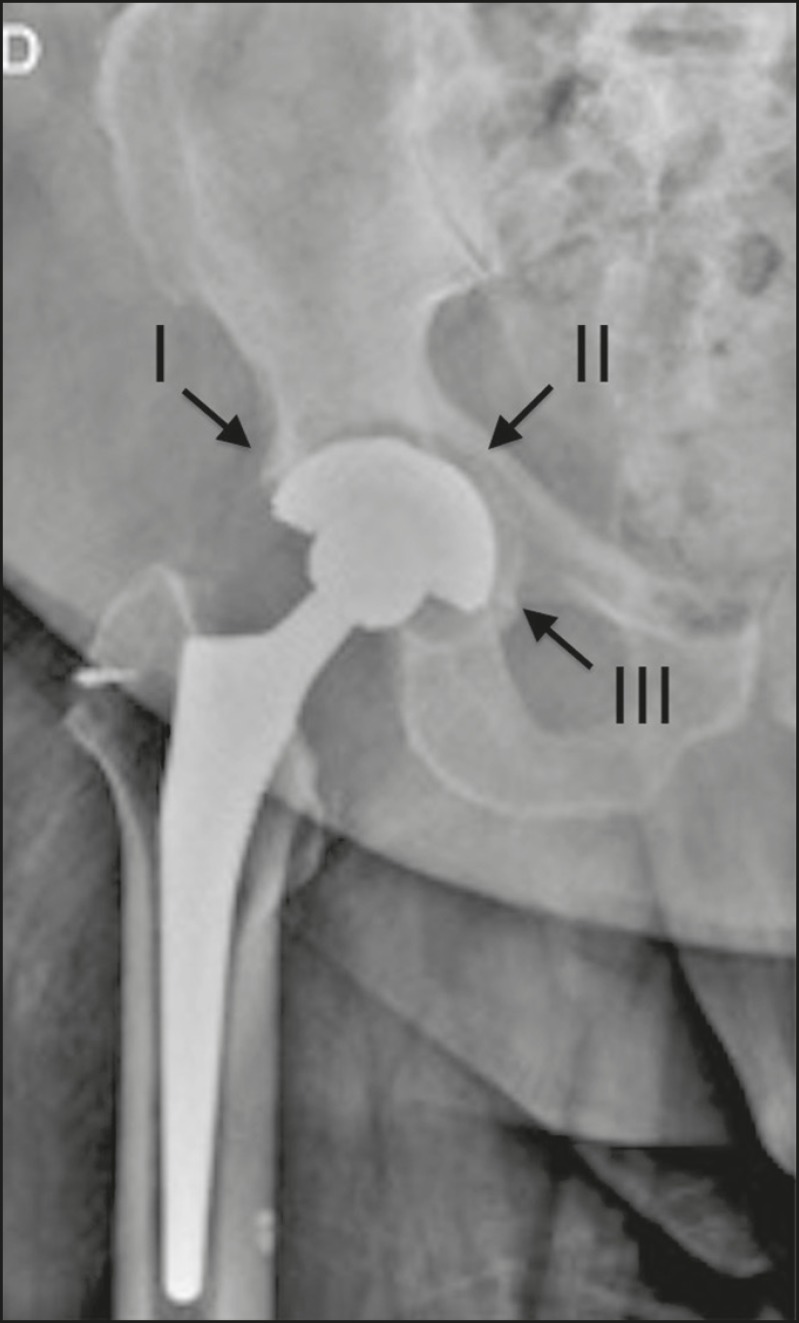
Anteroposterior X-ray of the right hip with osteolysis in zones I, II and III (arrows) of the right acetabular component.

**Figure 12:**
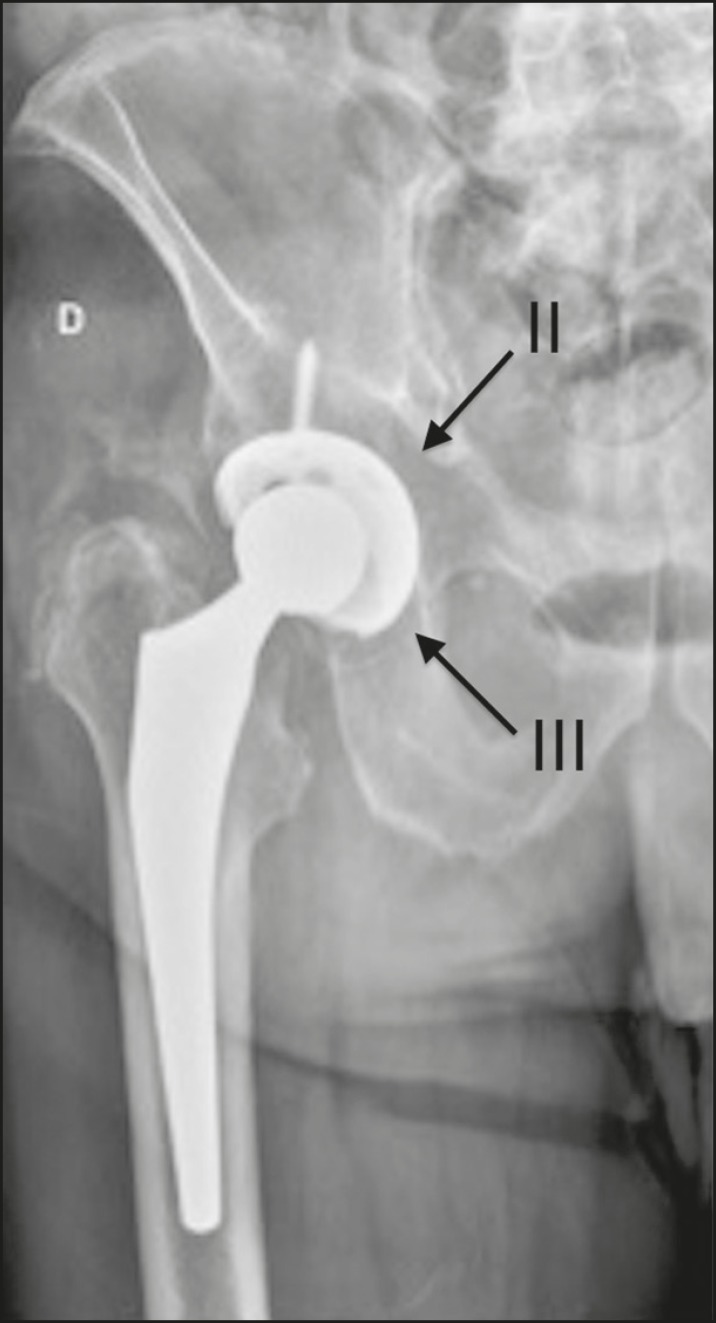
Anteroposterior X-ray of the right hip, showing osteolysis in acetabular zones II and III (arrows).

**Figure 13:**
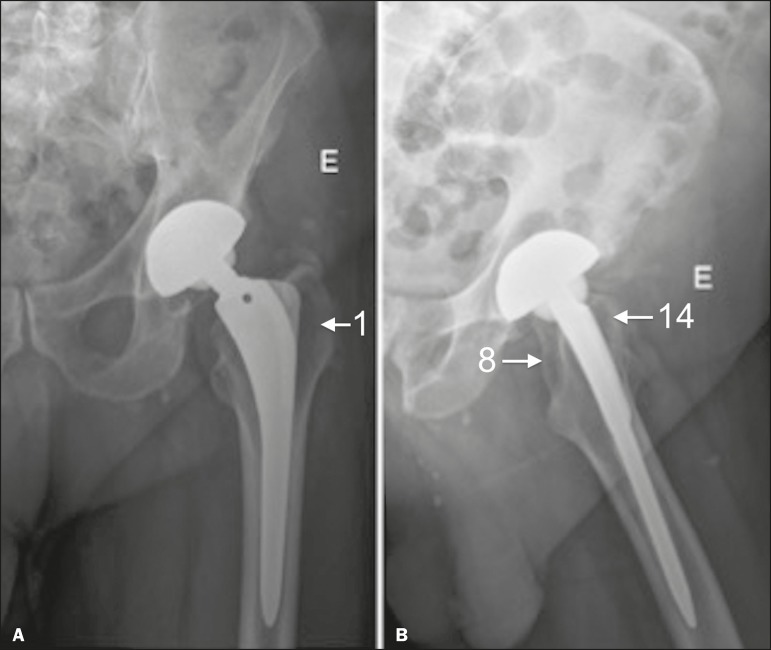
Anteroposterior and lateral X-rays of the left hip (**A** and **B**, respectively), showing osteolysis in femoral zones 1, 8, and 14 (arrows).

The description of the location of the foci of lucency should adhere to the standard orthopedic nomenclature for the femoral and acetabular zones^(7,8)^. The regions around the acetabular component are divided into three equal zones-I, II, and III-from lateral to medial. As for the femoral zones, there are seven in the anteroposterior view and another seven in the lateral view. In the anteroposterior view, the first three run proximal to distal along the lateral aspect of the femoral stem, zone 4 is at the tip of the stem, and zones 5-7 run distal to proximal along the medial aspect of the prosthesis. In the lateral view, zones 8 to 14 follow the same pattern, starting in the anterior and proximal aspect of the stem (Figure 10). We present some practical examples of femoral and acetabular osteolysis (Figures 11, 12, and 13).

## WEAR

**Figure 14:**
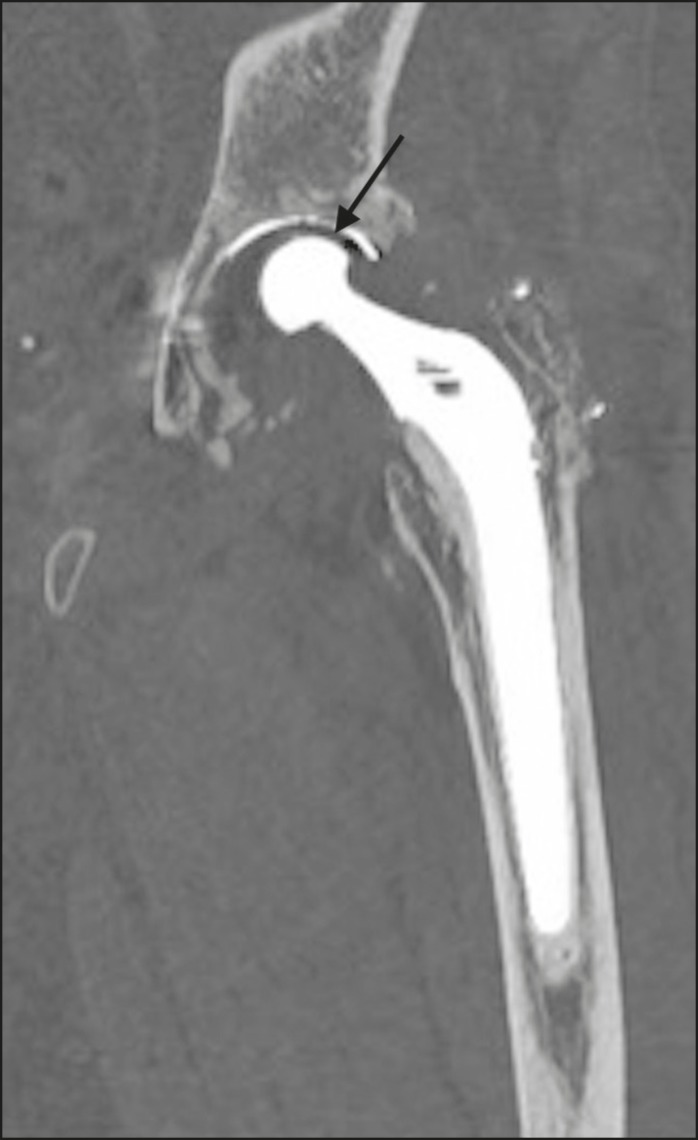
Computed tomography of the left hip in the coronal plane. Total left hip arthroplasty with reduction of the upper space, between the metallic head and acetabular components (arrow), indicating wear of the polyethylene lining.

**Figure 15:**
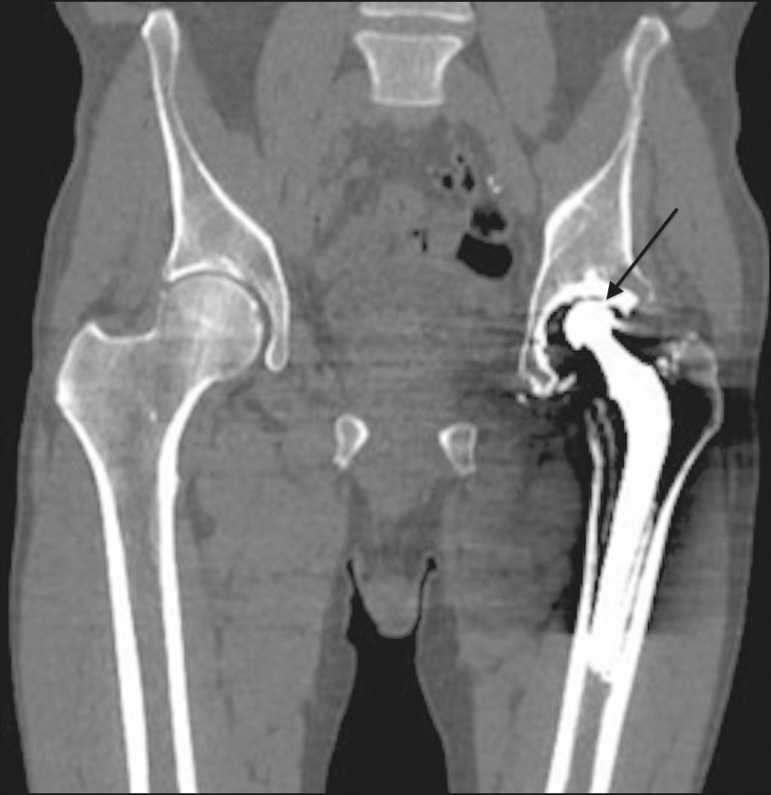
Coronal computed tomography scan showing a total left hip prosthesis, with asymmetry of the upper joint space, suggestive of wear of the polyethylene lining (arrow).

Wear typically occurs in hip arthroplasty involving the use of a prosthesis with a polyethylene liner. As depicted in Figures 14 and 15, the characteristic radiographic finding is asymmetrical positioning of the femoral head in the acetabular cup under load bearing^(8)^.

## DISLOCATION

**Figure 16:**
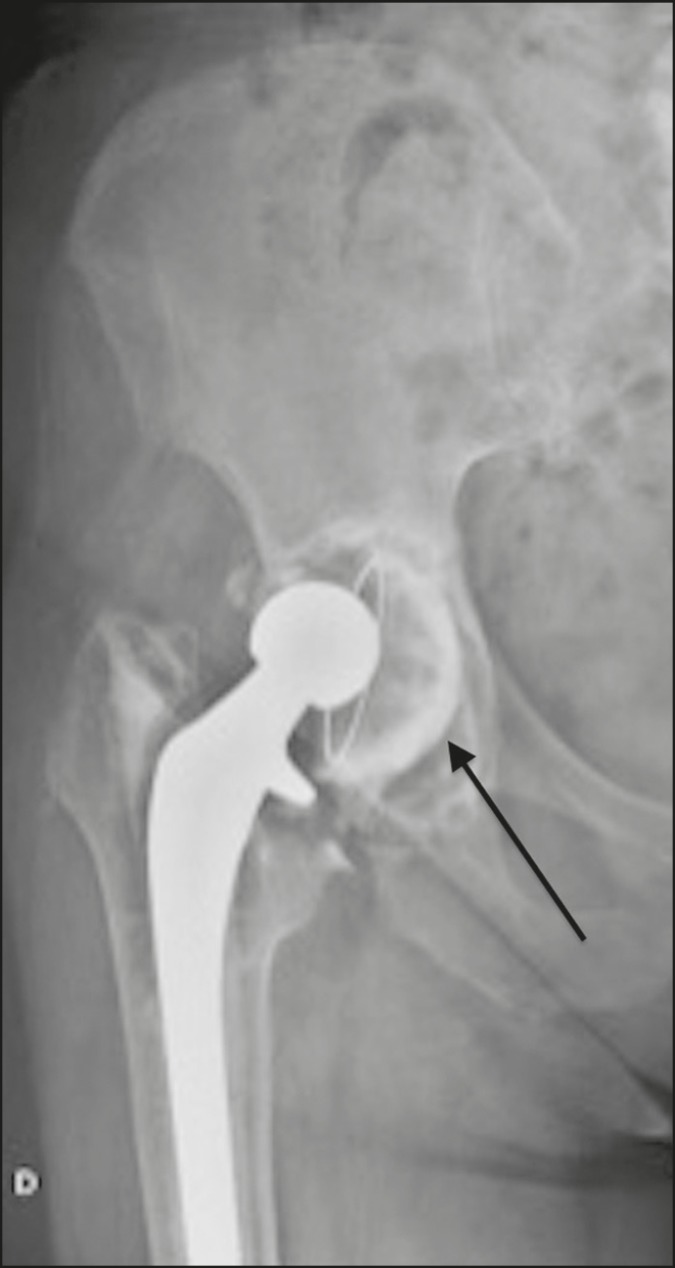
Anteroposterior X-ray of the right hip. Total right hip arthroplasty with vertical orientation and dislocation of the right acetabular component of the prosthesis (arrow).

Dislocation of the elements of a hip prosthesis is a major cause of surgical revision^(9)^. Within the first three months and more than five years after surgery, nontraumatic dislocation is usually due to laxity of the articular pseudocapsule and adjacent soft tissues, due to either immaturity (within the first three months) or lassitude (more than five years later). As can be seen in Figure 16, dislocation that occurs within the first three months or more than five years after surgery usually results from poor positioning of its components, such as the acetabular component, which can become vertically orientated, anteverted, or retroverted^(9)^.

**Figure 17:**
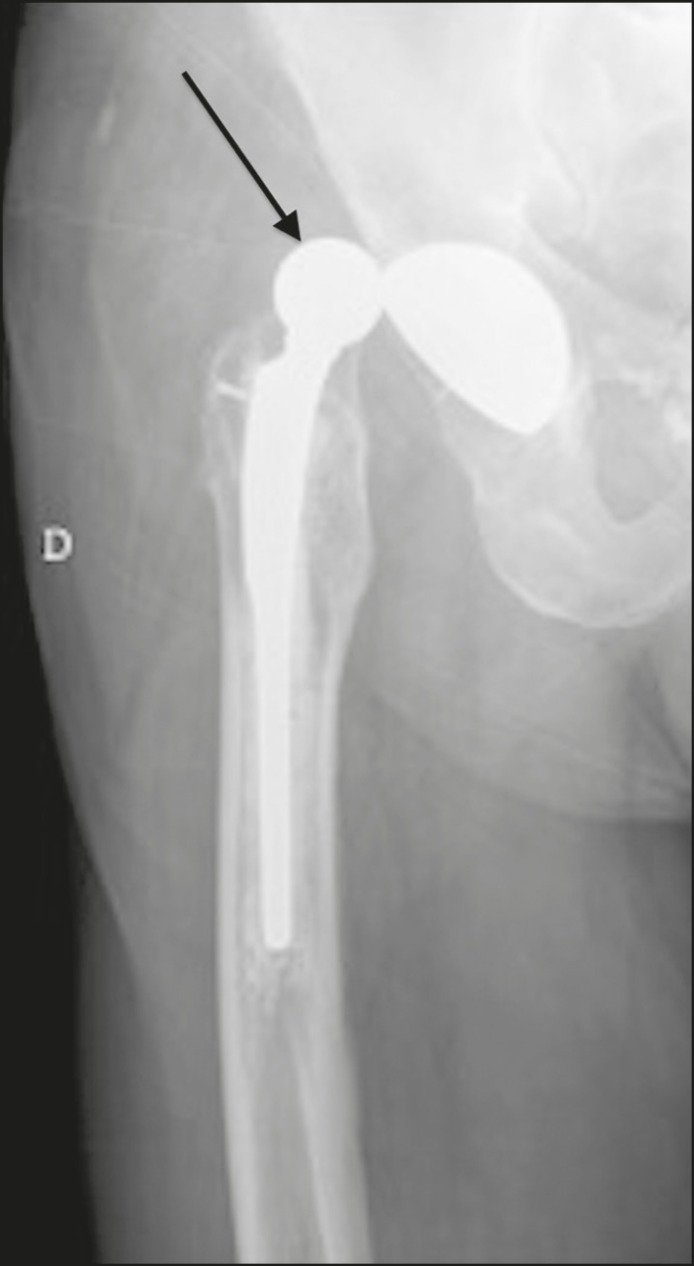
Anteroposterior X-ray of the right hip. Posterosuperior dislocation of the femoral component of right hip prosthesis (arrow), five years after the surgical procedure.

Early dislocations of prosthetic components are usually managed conservatively, whereas those occurring more than five years after arthroplasty usually require surgical management^(10)^, as depicted in Figure 17.

## CONCLUSION

The increased frequency of orthopedic surgical procedures have made it common for radiologists to encounter the potential complications. We hope this illustrative review will help our colleagues in radiology recognize the imaging patterns of some of the major complications of hip arthroplasty.
